# Comparison of lipid profiles and nutritional quality of 11 red-flowered oil-tea camellia through quantitative lipidomics and chemometrics

**DOI:** 10.1016/j.fochx.2025.102738

**Published:** 2025-07-04

**Authors:** Zhuang Deng, Jianmei Yang, Xinye Zhang, Chunyao Dun, Zhubing Hu, Guodong Wang, Qi Tang, Tao Zheng, Haitao Zeng

**Affiliations:** a School of Biological Science and Engineering, Shaanxi University of Technology, Hanzhong 723001, Shaanxi, China; b Hubei Academy of Forestry, Wuhan 430075, Hubei, China; c Enshi Tujia and Miao Autonomous Prefecture Academy of Forestry Sciences, Enshi 445000, China; d School of Life Sciences, Henan University, Kaifeng 475001, Henan, China; e College of Life Sciences, Engineering Research Center of High Value Utilization of Western China Fruit Resources of Ministry of Education, Shaanxi Normal University, Xi'an 710119, China; f Qinba State Key Laboratory of Biological Resources and Ecological Environment (Incubation), Shaanxi Key Laboratory of Bio-resources, Collaborative Innovation Center for Comprehensive Development of Biological Resources in Qinba Mountain Area of Southern Shaanxi, Hanzhong 723001, Shaanxi, China

**Keywords:** Red-flowered oil-tea camellia, Lipidomics, Nutritional quality, Chemometrics, Metabolic pathways

## Abstract

The aim of this study was to characterize the lipid profiles of 11 red-flowered oil-tea camellia (ROCs) using UPLC-MS/MS-based lipidomics. 618 lipids were identified, with TGs, PCs, PEs, PIs, DGs, and FFAs as the predominant lipid subclasses. TC1 exhibited the highest content of total lipids and GLs, followed by TC4 and DBXN. GPs were significantly elevated in TC1, TC2, TC3, and TS, whereas CC had the highest content of FFAs. CC, TS, DBXN, and TC lines exhibited unique lipid profiles suitable for functional product development. HCA and OPLS-DA demonstrated significant lipids variations among 11 ROCs, with 3 distinct clusters: CC-DBXN-XN, TC lines, and ZJ-GN-TS. Glycerophospholipid metabolism and glycerolipid metabolism were identified as key metabolic pathways, with LPC(18:2), PA(18:2_18:2), PA(16:0_18:2), PC(18:3_18:3), and TG(18:1_18:2_18:4) serving as characteristic differential lipids across 11 ROCs. Our findings provided insights into quantitative differential lipids as potential biomarkers for directional breeding and functional product development across ROCs.

## Highlights

• 618 lipids were identified in 11 red-flowered oil-tea Camellia (ROCs).
• Significant lipids variations revealed 3 distinct clusters: CC-DBXN-XN, TC lines, and ZJ-GN-TS.
• 5 differential lipids served as characteristic differential lipids across 11 ROCs.
• Glycerophospholipid metabolism and glycerolipid metabolism were the key metabolic pathways.
• The core regulatory nodes of lipids in 11 ROCs comprised DGs, TGs, FFAs, PAs, PCs, PEs, and PIs.

## 1 Introduction

Camellia oil is widely recognized as a high-quality edible oil owing to its exceptional nutritional composition. Red-flowered oil-tea camellias (ROCs) have emerged as economically important crops with superior nutritional value (Zhong et al., 2023). Compared to the more common *Camellia oleifera* Abel., ROCs demonstrate greater economic potential and development prospects due to its distinctive oil quality and vibrant coloration (Zeng, Zheng, et al., 2024). Although ROCs occupy smaller cultivation areas in China than *C. oleifera*, they generally exhibit higher seed oil content. This characteristic, combined with their excellent oil quality, has established ROCs as crucial germplasm resources for *Camellia* varietal improvement. The most common ROCs species included *Camellia chekiangoleosa*, *Camellia semiserrata*, *Camellia taishunensis*, *Camellia reticulata*, and *Camellia pitardii* (Wang et al., 2022). These ROCs varieties had attracted considerable research attention for their oil-rich seeds, which were extensively utilized in both edible oil production and functional food development (Zheng et al., 2024). The unique lipid composition of these ROCs seeds not only determines their nutritional quality and oxidative stability but also confers various potential health advantages, comprising cardioprotective properties and significant anti-inflammatory activity. The growing interest in these species reflects their increasing importance in the edible oil industry and health food markets.

Lipids serve as the primary nutritional constituents of the camellia seed oil, playing a pivotal role in determining its quality and oxidative stability (Zhang, Zhu, et al., 2022). The nutritional value and organoleptic properties of camellia oil are fundamentally influenced by both the content and composition of key lipid components, including fatty acids (FAs), triacylglycerols (TGs), and glycerophospholipids (GPs) (Dong et al., 2025). Notably, the nutritional quality of camellia oil demonstrated a significant association with its fatty acid profile, particularly the proportion of unsaturated fatty acids, which was significantly contributed to both its oxidative stability and conferring health-promoting properties (Yang et al., 2025). Despite the critical importance of these lipid components, comprehensive comparative analyses of lipid profiles among different ROCs varieties remained limited. The current lack of in-depth researches on lipid composition in ROCs, coupled with insufficient understanding of lipid functionality, has emerged as a major constraint in the promotion of superior camellia cultivars and the development of premium camellia oil products. Consequently, precise characterization of the composition and relative content of lipids in ROCs was imperative for elucidating their biological functions and assessing their nutritional and health-promoting qualities.

Lipidomics, as an essential branch of metabolomics, displays a pivotal role in elucidating the functional properties and distinctive lipid profiles within foods (Zeng, Liu, et al., 2024). The integration of advanced UPLC-MS/MS techniques with chemometric approaches had established lipidomics as an indispensable methodology for comprehensive characterization and precise identification of intricate food matrices, particularly for lipids with low-abundance (Hu et al., 2021; Theodoridis et al., 2010). In our previous investigations, lipidomic profiling of 23*C. oleifera* lines from southern Shaanxi delineated their lipid composition and enabled the selection of 7 superior lines (Zheng et al., 2024). Furthermore, a comparative lipidomic analysis of the rapeseed variety ‘Fangyou 777’ and its parental lines uncovered dynamic lipid alterations across different developmental stages (Zheng et al., 2025). Notably, phosphatidic acid (PAs), lysophosphatidic acid (LPAs), and diacylglycerol (DGs) were identified as critical lipid mediators during coffee bean maturation through differential lipidomic profiling (Wang et al., 2024). Quantitative lipidomic assessment of rambutan seed oils revealed that 807 lipids were identified, with TGs, DGs, and PEs being the predominant ones (Cui, Zhao, et al., 2024). Lipidomics studies revealed that phosphatidylcholine (PCs) and phosphatidylethanolamine (PEs) were the major phospholipids in oat and wheat varieties, exhibiting similar abundance and gradual decreases during endosperm development (Li et al., 2024). Despite these methodological and analytical advancements, a systematic exploration of lipid characteristics across different ROCs remained conspicuously absent in the literature, representing a critical knowledge gap in current lipidomic research.

Comprehensive lipidomic profiling of 11 ROCs was conducted through lipidomics based on UPLC-MS/MS to elucidate their lipid composition and functional characteristics. Chemometric approaches (PCA, OPLS-DA, and HCA) were employed to assess lipid variations among the ROCs and identify differentially expressed lipids. This study provided a robust scientific foundation for the quantitative differential lipids and practical applications of camellia seed oil, facilitated informed dietary lipid intake, and contributed to identifying novel biomarker candidates in lipids of ROCs.

## 2 Materials and methods

### 2.1 ROCs fruits samples collection

A total of 11 ROCs lines were collected during mid-October 2024. The collection encompassed 3 distinct geographical sources: (1) Qinba Mountain specimens including *C. chekiangoleosa* (ZJ), *C. taishunensis* (TS), and *C. reticulata* (TCHZ); (2) Wuling Mountain varieties comprising *Camellia qrossideulata* Chang Sect. (CC), *C. pitardii* var. compressa (DBXN), *C. pitardii* (XN), and *C. semiserrata* (GN); and (3) 4*C. reticulata* lines (TC1-TC4) obtained from Tengchong City, Yunnan Province. For each sample, approximately 2.0 kg of mature fruits were systematically harvested from multiple canopy directions of selected healthy trees to ensure representative sampling. The collected fruits underwent natural desiccation through shade-drying at ambient temperature (25 ± 3 °C) until reaching constant mass.

### 2.2 Sample preparation and lipids extraction

All ROC samples underwent freeze-drying followed by mechanical pulverization. 20 mg of the ground dry sample was weighed and aliquoted into a 2.0 mL centrifuge tube containing 1 mL of the extraction solvent (MTBE: MeOH =3:1, *v*/v). The mixture was oscillated at 2500 r/min for 15 min at room temperature. Subsequently, phase separation induction with 300 μL of ultrapure water, the solution was vortex-mixed for 1 min, and allowed to stand at 4 °C for 10 min. Phase separation was achieved through centrifugation at 12,000 rpm (4 °C, 10 min). 200 μL of the supernatant was transferred to a 1.5 mL centrifuge tube and dried completely by concentration at 20 °C for 2.0 h. The dried residue was reconstituted with 200 μL lipid reconstitution solution (acetonitrile: isopropanol = 1:1, *v*/v), vortexed for 3.0 min, and centrifuged at 12,000 r/min for 3.0 min (4 °C). Finally, 120 μL of the supernatant was aliquoted into an injection vial for UPLC-MS/MS analysis (Zheng et al., 2024; Zheng et al., 2025).

### 2.3 Lipids determination

Lipidomic analysis of 11 ROCs lines was conducted through an UPLC system (UPLC, ExionLC AD, AB SCIEX) coupled with a Thermo Accucore™C30 column (2.6 μm, 2.1 mm × 100 mm i.d.). The analytical conditions were as follows, solvent system, A: acetonitrile/water (60/40, *V*/V, 0.1 % formic acid, 10 mmol/L ammonium formate), B: acetonitrile /isopropanol (10/90, V/V, 0.1 % formic acid, 10 mmol/L ammonium formate); gradient program (*V*/V), A/B (80:20, V/V) at 0 min; A/B (70:30, V/V) at 2.0 min; A/B (40:60, V/V) at 4.0 min; A/B (15:85, V/V) at 9.0 min; A/B (10:90, V/V) at 14.0 min; A/B (5:95, V/V) at 15.5 min; A/B (5:95, V/V) at 17.3 min; A/B (80:20, V/V) at 20.0 min; flow rate, 0.35 mL/min; column temperature, 45 °C; injection volume, 2.0 μL. The effluent was alternatively connected to an ESI-triple quadrupole-linear ion trap (QTRAP)-MS for detection (Zheng et al., 2024).

Mass spectrometric (MS) analyses were performed using a QTRAP® 6500+ LC-MS/MS System (AB SCIEX) equipped with an electrospray ionization (ESI) Turbo Ion-Spray interface. The instrument was operated in both positive and negative ionization modes under the control of Analyst 1.6.3 software (AB SCIEX). The MS analysis was conducted under optimized parameters, including (1) Electrospray ionization (ESI) source temperature at 500 °C; (2) ion spray voltage of 5500 V in positive ion mode and − 4500 V in negative ion mode; (3) ion source gas 1 (GS1) at 45 psi, gas 2 (GS2) at 55 psi, and curtain gas (CUR) at 35 psi. Instrument tuning and mass calibration were performed with 10 and100 μmol/L polypropylene glycol solutions in QQQ and LIT modes, respectively. QQQ scans were acquired as MRM experiments with collision gas (nitrogen) set to 5 psi. In the triple quadrupole system, each ion pair is scanned and detected according to the optimized declustering potential (DP) and collision energy (CE).

### 2.4 Lipids identification and quantification analysis

The MS data files were processed using Analyst 1.6.3 software (AB SCIEX). Qualitative identification of lipids in the 11 studied ROCs was carried out through high-resolution MS analysis combined with self-built MetWare Database (MWDB), incorporating characteristic retention times and precursor ion *m*/*z* values. For quantitative analysis, lipid species were measured using Multiple Reaction Monitoring (MRM, **Fig.S1**) mode on a Triple Quad 6500^+^ system (AB SCIEX). The content of each lipid was quantitatively analyzed using the internal standard method (**Table S1**). The content of lipid compounds (X, in μg/g) was calculated according to the following formula: X = 0.000001*R*c*F*V*M/m (X: Lipid compound content, μg/g; R: Ratio of the chromatographic peak area of the target compound to the chromatographic peak area of the internal standard; c: Internal standard concentration, μmol/L; F: Internal standard correction factor; V: Volume of the sample extract, μL; M: Precise mass; m: Sample quality, g).

Following the acquisition of lipid MS data across 11 ROCs samples, chromatographic peaks integration and alignment were rigorously performed for each detected lipids to ensure quantification precision.

### 2.5 Statistical analysis

Lipid profile data for all ROCs samples, analyzed in triplicate, were expressed as mean ± standard deviation. Comparative analysis of lipids content across the 11 ROCs was performed using one-way ANOVA followed by Duncan's test, implemented in SPSS 26.0 (IBM Corp., Armonk, NY, USA). Differential lipid identification among the 11 ROCs was conducted through OPLS-DA method, employing stringent selection criteria of VIP > 1 combined with a fold change (FC) threshold of >2 or < 0.5. Comprehensive data visualization was subsequently executed using OriginPro 2025b (OriginLab, Northampton, MA, USA), incorporating multiple analytical representations: f histogram, bubble diagram, clustering bar chart, combined heatmap, Venn diagrams for feature intersection analysis, and OPLS-DA score plots to illustrate group separations.

## 3 Results

### 3.1 Lipid profiles of 11 ROCs samples

**Fig. 1:**
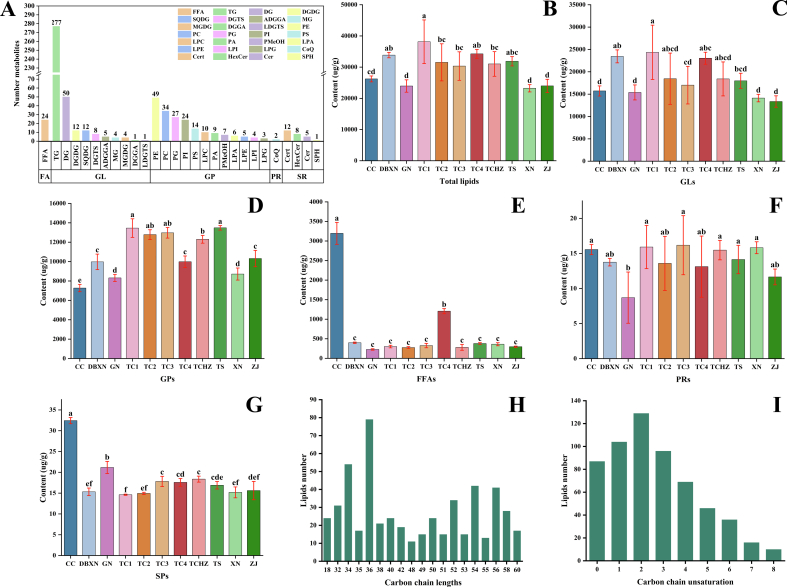
Lipids profiles of 11 ROCs samples. **A**: Lipids categories and subclasses. **B**: All lipids content. **C**: GLs content. **D**: GPs content. **E**: FAs content. **F**: PRs content. **G**: SPs content. **H**: Lipid carbon chain lengths distribution histogram. **I**: Lipid carbon chain unsaturation distribution histogram. The different lowercase letters indicated statistically significant differences at the 0.05 level.

The highly consistent total ion current (TIC) profiles obtained in both positive ion mode (P+) (**Fig. S2A**) and negative ion mode (N-) (**Fig. S2B**) during MS analysis demonstrated the stability and reliability of lipids data. A total of 618 lipid molecules were preliminarily identified and classified into 5 categories, comprising 374 GLs, 192 GPs, 26 SPs, 24 FAs, and 2 PRs (Fig. 1A **and Table S2**). These 618 lipids were further divided into 28 subclasses, comprising 277 TGs, 50 DGs, 49 PEs, 34 PCs, 27 PGs, 24 FFAs, 24 PIs, 14 PSs, 12 DGDGs, 12 SQDGs, 12 Certs, 10 LPCs, 9 PAs, 8 DGTSs, 8 HexCers, 7 PMeOHs, 6 LPAs, 5 LPEs, 5 ADGGAs, 5 Cer, 4 MGs, 4 MGDGs, 4 LPIs, 3 LPGs, 2 CoQs, 1 DGGA, 1 LDGTS, and 1 SPH. Notably, the lipid profile of ROCs demonstrated abundant compositional diversity, particularly in GLs, with TGs constituting a significant proportion, and also contained unique components such as SPs and PRs, indicating its benefits in promoting energy expenditure, inhibiting fat accumulation, and preventing hypertension-related diseases. Our findings highlighted the rich health properties and promising development potential of ROCs lipids.

From the perspective of major lipid classification, GLs emerged as the predominant lipid class, serving as the primary structural foundation of the lipid composition. Notably, GLs, FAs, and GPs collectively formed the 3 major lipid fractions that dominated the lipid profile. The total lipids content varied significantly among 11 ROCs samples (*p* < 0.05). TC1 exhibited the highest lipids content (38,149.47 ± 5701.10 μg/g), followed by TC4 (34,261.12 ± 1154.83 μg/g) and DBXN (33,854.43 ± 709.18 μg/g) (Fig. 1B). The distribution pattern of GLs closely mirrored that of total lipid content, demonstrating a consistent proportional relationship throughout the analysis (Fig. 1C). The GPs content was significantly higher in TC1, TC2, TC3, and TS compared to other varieties, with the TC lines showing particularly pronounced elevations (Fig. 1D). In contrast, the highest FAs levels were detected in CC samples (3195.33 ± 228.59 μg/g) (Fig. 1E). PRs exhibited the lowest average content among all lipid classes, with no obviously significant inter-varietal differences (Fig. 1F). Conversely, SPs were most abundant in CC samples, reaching 32.43 ± 0.60 μg/g (Fig. 1G).

From the perspective of lipid subclass distribution, the three most abundant subclasses were TGs, PCs, and PEs, followed by PIs, DGs, MGs, LPCs, and PAs in decreasing abundance. The remaining 20 lipid subclasses collectively accounted for minor proportions, revealing substantial heterogeneity in abundance across the 28 analyzed subclasses. Notably, TGs exhibited exceptionally high concentrations exceeding 20,000 μg/g in DBXN, TC1, and TC4 samples. Elevated levels of PCs and PEs were observed in both TC lines and TS samples, with TC lines demonstrating particularly prominent accumulation: mean PCs concentrations reached 5651.55 ± 673.53 μg/g, while PEs levels averaged 3717.32 ± 381.94 μg/g.

Due to the importance of carbon chain length and saturation in lipids, these characteristics were systematically analyzed in 618 identified lipids. Our analysis revealed that the carbon chain length of the lipids ranged from 10 to 62, with medium- to long-chain lipids (32–58 carbons) accounting for approximately 85.76 %. Studies have shown that medium- and long-chain lipids could reduce body weight and fat content, promote energy expenditure, and might be developed into functional health foods with specific benefits. The degree of unsaturation in carbon chains ranged from 0 to 14, with the most common being 2 (129, 20.87 %), 1 (104, 16.83 %), 3 (96, 15.53 %), and 4 (69, 11.17 %). Unsaturated lipids (with a lipid unsaturation degree greater than 0) accounted for approximately 85.60 %. Unsaturated lipids serve as key mediators in multiple physiological processes, including blood lipid regulation, thrombosis prevention, and visual fatigue alleviation. Consequently, the structural characteristics of ROC lipids, particularly carbon chain length and degree of unsaturation, emerged as critical determinants for their health-promoting biological activities, manifesting in diverse pharmacological effects ranging from hypolipidemic and antidepressant actions to antioxidant activity and antitumor properties.

### 3.2 Nutritional quality assessment

The nutritional quality of ROCs was evaluated based on the fatty acid composition, lipid class distribution, and the presence of bioactive compounds.

**Fig. 2:**
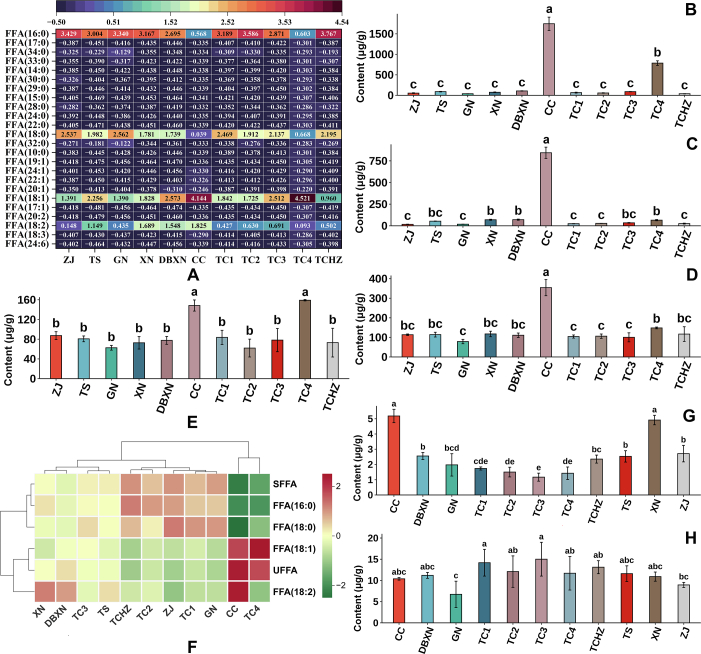
**A:** Heatmap analysis of FFAs across 11 ROCs. **B-E:** Comparison of the contents of FFA(18:1), FFA(18:2), FFA(16:0), and FFA(18:0) across of 11 ROCs, respectively. **F:** Heatmap analysis of the proportions of FFA(18:1), FFA(18:2), FFA(16:0), and FFA(18:0). **G-H:** Comparison of the contents of CoQ 9 and CoQ10 across of 11 ROCs. The different lowercase letters denoted statistically significant differences at the 0.05 level.

Among the 11 ROCs samples, 24 distinct FFAs were detected, and the predominant FFAs identified were composed of FFA(18:1), FFA(18:2), FFA(16:0), and FFA(18:0) (Fig. 2A). Notably, the CC samples exhibited the highest content of FFA(18:1) (1746.20 ± 161.22 μg/g), followed by TC4 samples (785.75 ± 56.13 μg/g) (Fig. 2B). Similarly, the CC samples showed the highest levels of FFA(18:2) (843.27 ± 64.96 μg/g; Fig. 2C) and FFA(16:0) (354.26 ± 41.34 μg/g; Fig. 2D). Additionally, FFA(18:0) content was significantly higher in both TC4 and CC samples compared to other ROCs samples (Fig. 2E). Collectively, the CC and TC4 samples demonstrated markedly higher content of these 4FFAs relative to the other ROC samples. The clustering heatmap results revealed that TC4 and CC had an unsaturated free fatty acid (UFFA) content exceeding 70 %, classifying them as high UFFA groups. In contrast, TCHZ, ZJ, TC1, GN, and TC2 exhibited a saturated free fatty acid (SFFA) content above 67 %, belonging to the high SFFA category. The remaining samples, TS, XN, DBXN, and TC3, exhibited intermediate characteristics, clustering between these 2 distinct groups (Fig. 2F). The UFFA/SFFA ratio was highest in CC (5.05), followed by TC4 (2.65), indicating a more favorable nutritional profile. Importantly, a higher UFFA/SFFA ratio was correlated with a reduced risk of cardiovascular diseases. FFA(18:1) demonstrated beneficial effects by reducing atherosclerosis, improving lipid metabolism, and enhancing wound healing. Conversely, excessive FFA(18:2) levels might promote inflammatory responses, potentially increasing risks of cardiovascular diseases, arthritis, and related disorders (Koundouros et al., 2025). These findings suggested that ROCs with high FFA(18:1) and low FFA(18:2) content represented the nutritionally optimal choices. Notably, the cluster heatmap identified TC4 as possessing the most favorable fatty acid profile, characterized by the highest FFA(18:1) content (65.12 %) and the lowest linoleic acid FFA(18:2) concentration (5.41 %) (Fig. 2F). This distinctive composition established TC4 as the superior variety for edible oil production from both nutritional and health perspectives.

With distinct compositional profiles and functional properties, CC samples demonstrated significant cardioprotective effects and the capacity to mitigate cardiovascular diseases. These benefits, along with potential anti-tumor, anticancer, antioxidant, and anti-inflammatory activities, might be attributed to their high concentrations of FAs and SPs (Fig. 1E **and** Fig. 1G). In contrast, TC1, TC2, TC3, TCHZ and TS samples were abundant in GPs and SPs, exhibiting potential in reducing cholesterol levels and enhancing cognitive and brain functions. Furthermore, TC1, TC4, and DBXN samples, characterized by their elevated GLs content, could be served as efficient energy sources due to their metabolic properties. The coenzyme Q9 (CoQ9) content was highest in CC and XN, with 5.18 ± 0.43 μg/g and 4.91 ± 0.31 μg/g, respectively (Fig. 2G). The TC samples contained relatively high levels of Coenzyme Q10, with TC1 and TC3 showing significantly higher concentrations than other samples, at 15.01 ± 3.98 μg/g and 14.18 ± 3.15 μg/g, respectively (Fig. 2H). The clinical trials had been verified that CoQ10 was a crucial coenzyme in humans, playing a central role in energy production and antioxidation. It has well-documented clinical value and is used as adjuvant therapy for cardiovascular diseases (e.g., heart failure) and neurodegenerative disorders (e.g., Parkinson's disease) (Zheng et al., 2024).

The analytical results revealed distinct functional advantages across 11 ROCs lines. Specifically, TC4 emerged as superior candidates for edible oil applications, characterized by their favorable fatty acid profiles featuring higher FFA (18:1) and lower FFA content. Notably, the TC1, TC2, TC3, TCHZ and TS demonstrated significant potential for immune-enhancing functional food development, primarily attributable to their abundant GPs content. From a nutraceutical perspective, TC1 and TC3 showed particular promise as candidates for developing dietary interventions targeting hypertension prevention and metabolic syndrome management, based on their exceptional CoQ10 concentrations.

### 3.3 Chemometrics-based discrimination of lipidomic signatures

**Fig. 3:**
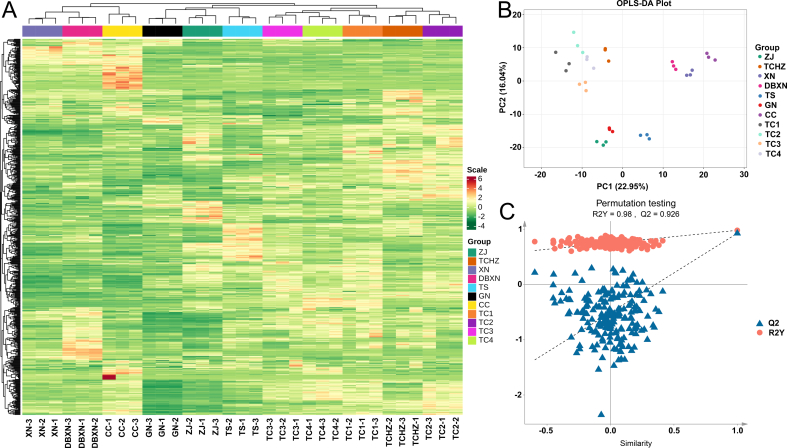
**A:** Hierarchical clustering heatmap. **B:** OPLS-DA score plot. **C:** permutation test of the OPLS-DA model.

To comprehensively compare the lipidomic profiles and identify differentially abundant lipids among the 11 ROCs samples, systematic multivariate statistical analyses were conducted. HCA with heatmap visualization revealed substantial variations in lipid composition, demonstrating clear segregation patterns across the samples that indicated pronounced inter-group heterogeneity (Fig. 3A). Notably, the analysis delineated 3 primary clusters: the CC-XN-DBXN samples formed a distinct group; the TC lines constituted a separate cluster; while ZJ-TS-GN samples were grouped together in a third cluster.

The lipidomic variations identified by HCA were further substantiated through OPLS-DA. This advanced multivariate approach enhanced inter-group discrimination, and identified key discriminatory lipid metabolites that critically influenced the oil quality characteristics of 11 ROCs samples. These complementary analytical approaches offered potential optimization strategies for the visualization of lipids differences across the 11 ROCs. Importantly, the OPLS-DA results strongly supported the HCA findings, with both methods consistently classifying the 11 samples into 3 well-defined groups (Fig. 3B). The OPLS-DA model demonstrated exceptional statistical reliability, as confirmed by rigorous permutation testing (R^2^Y = 0.98, Q^2^ = 0.926), indicating both superior model fit and robust predictive performance (Fig. 3C).

Accordingly, our subsequent analysis focused on identifying differential lipids both among these 3 classified groups and across all 11 ROCs samples.

### 3.4 The differential lipids among XN, DBXN, and CC samples

Based on the OPLS-DA results, highly differential lipids were identified through the criteria of VIP > 1 and *p*-value <0.05. 202 differential lipids were identified among CC, DBXN, and XN (**Table S3**), which were distributed across 4 major lipid classes (SPs, *N* = 14; GPs, *N* = 47; GLs, *N* = 123; FAs, *N* = 18), indicating that different varieties significantly influenced lipid diversity.

**Fig. 4:**
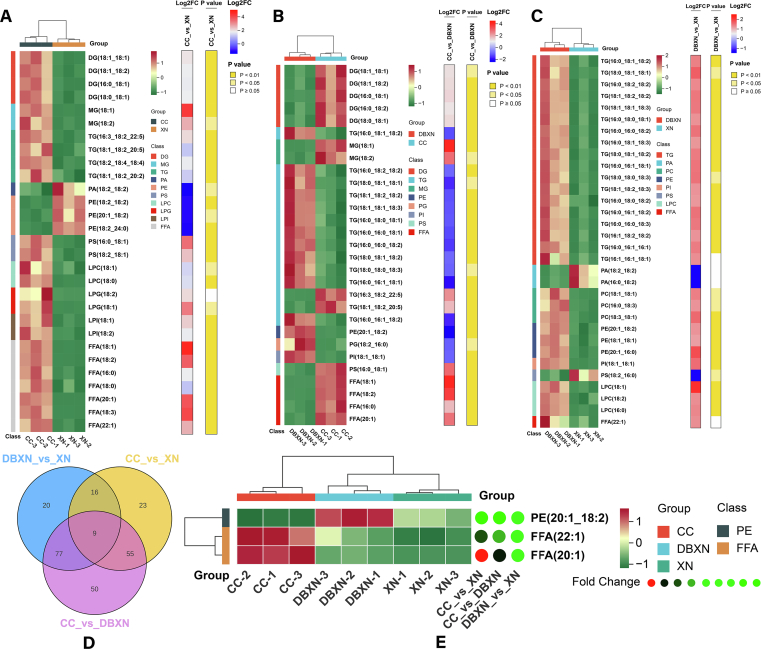
**A-C:** The combined heatmap of differential lipids with higher content in CC vs XN comparison, CC vs DBXN comparison, and DBXN vs XN comparison. **D:** Veen plot. **E:** Hierarchical clustering of 3 characteristic differential lipids.

In the CC_vs_XN comparison, 103 differentially abundant metabolites were identified, comprising 72 significantly upregulated and 31 downregulated (**Table S4**). Lipidomic analysis revealed that the predominant differentially expressed lipids belonged to GLs, GPs, and FFAs (Fig. 4A). These metabolites were primarily enriched in biosynthesis of unsaturated fatty acids, glycerophospholipid metabolism, and fatty acid biosynthesis. Notably, FFAs demonstrated significant elevation in CC samples, particularly unsaturated fatty acids including FFA(18:1) (1746.20 ± 131.63 μg/g, 23.53-fold increase), FFA(18:2) (843.27 ± 53.04 μg/g, 12.09-fold), and FFA(18:3) (15.38-fold), indicating substantial accumulation in CC samples relative to XN samples. The CC samples also exhibited marked increases in 3 DGs [DG(18:1_18:1), DG(18:1_18:2), and DG(16:0_18:1)] and 2 MGs [MG(18:1) and MG(18:2)] compared to CN controls. Among GPs, 2 PSs [PS(16:0_18:1) and PS(18:2_18:1)] exhibited pronounced upregulation with 10.14- and 4.67-fold, respectively. Conversely, PEs and PAs, including PE(18:2_18:2), PE(20:1_18:2), and PA(18:2_18:2), were significantly downregulated in CC samples.

The CC_vs_DBXN comparison identified 191 differential lipids, with 81 upregulated and 110 downregulated (**Table S5**). The lipid profile was similarly dominated by GLs, GPs, and FFAs (Fig. 4B). FFAs levels remained significantly elevated in CC samples, particularly FFA(18:1) (16.35-fold), FFA(18:2) (11.90-fold), and FFA(20:1) (15.38-fold). Consistent with the previous comparison, 3 DGs and 2 MGs showed increased abundance in CC relative to DBCN controls. Notably, 3 TGs [TG(16:0_18:1_18:2), TG(16:0_18:2_18:2), and TG(18:0_18:1_18:1)] demonstrated substantial upregulation in DBXN samples (2.52-, 2.02-, and 3.22-fold, respectively). GPs samples revealed significant downregulation of PE(20:1_18:2), PG(18:2_16:0), and PI(18:1_18:1) in CC samples.

In the DBXN_vs_XN comparison, 122 differential metabolites were identified (97 upregulated, 25 downregulated), predominantly comprising GLs and GPs (**Table S6**). TGs showed remarkable accumulation in DBXN samples, particularly TG(16:0_18:1_18:2) (2651.94 ± 254.48 μg/g, 2.42-fold), TG(18:0_18:1_18:1) (2142.26 ± 291.84 μg/g, 3.01-fold), TG(16:0_18:2_18:2) (1789.81 ± 115.14 μg/g, 2.27-fold), TG(18:1_18:2_18:2) (1529.62 ± 115.64 μg/g, 2.58-fold), and TG(18:1_18:1_18:3) (1380.35 ± 115.48 μg/g, 2.54-fold) (Fig. 4C). Additionally, 3 LPCs [LPC(18:1), LPC(18:2), and LPC(16:0)], PC(18:1_18:1), PE(20:1_18:2), and PI(18:1_18:1) were significantly elevated in DBXN samples compared to CN controls. Conversely, 2 PAs [PA(18:2_18:2), PA(16:0_18:2)] and PS(18:2_16:0) showed higher abundance in XN samples.

The Venn diagram analysis revealed 9 common lipids that were differentially expressed across all 3 comparisons (Fig. 4D). Among these 9 shared lipids, PE(20:1_18:2), FFA(20:1), and FFA(22:1) exhibited higher content. Notably, PE(20:1_18:2) showed predominant elevation in DBXN samples, whereas both FFA(20:1) and FFA(22:1) were significantly enriched in CC samples (Fig. 4E). HCA results demonstrated clear segregation of the 3 groups based on these lipid profiles, indicating their potential utility as distinctive molecular markers for differentiating the 3 ROCs.

### 3.5 The differential lipids among TC lines

**Fig. 5:**
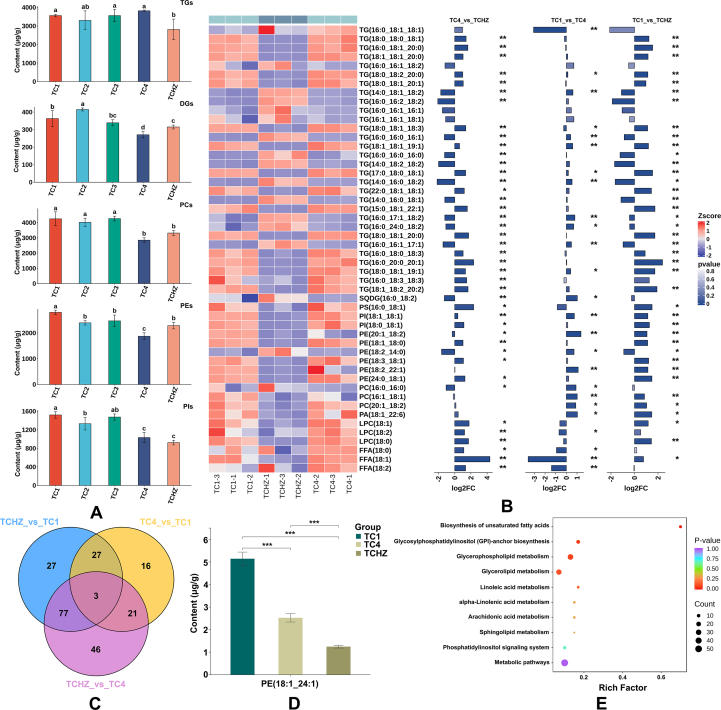
**A:** Comparison of the contents of TGs, DGs, PCs, PEs, PIs, respectively. **B**: Differential lipids with higher content among TC1, TC4, and TCHZ. **C**: Veen plot. **D**: Bar plot of PE(18:1_24:1). **E**: KEGG pathway.

Based on the criteria of VIP > 1 and *p*-value <0.05 for screening significantly differential lipid compounds, a total of 191 significantly altered lipids across the TC lines were identified (*p* < 0.05), categorized into 5 main lipid subclasses: TGs, DGs, PCs, PIs, and PEs (**Table S7**). To comprehensively visualize the variations among these 5 lipid subclasses in different TC lines, we generated differential abundance histograms through multivariate analysis, which revealed statistically significant differences (*p* < 0.05) in lipid profiles among TC1, TC4, and TCHZ samples compared to other TC lines (Fig. 5A).

Based on these findings, we specifically selected these 3 distinct samples (TC1, TC4, and TCHZ) for subsequent detailed comparative analysis to elucidate intra-lineage variations of lipids within the TC lines. A total of 217 lipids with significant differences were identified (VIP >1, *p* < 0.05, FC >2.0 or < 0.50), while the TC4 displayed upregulation of 32 lipids and downregulation of 35 lipids compared to TC1 (**Table S8**). When comparing TCHZ with TC1, we observed 45 upregulated and 89 downregulated lipids (**Table S9**). TCHZ exhibited substantial lipid profile alterations compared to TC4, with 65 upregulated and 82 downregulated lipids (**Table S10**). Statistical analysis of lipid profiles revealed that the differential lipids with higher content were found in TGs, PIs, and FFAs, followed by PEs and PCs. 3 TGs [TG(18:0_18:0_18:1), TG(16:0_18:1_20:0), TG(16:0_18:1_20:0)] showed the lowest levels in TCHZ and the highest content in TC4. 5 TGs [TG(14:0_18:1_18:2), TG(16:0_16:2_18:2), TG(16:0_16:0_16:0), TG(14:0_18:2_18:2), TG(14:0_16:0_18:1)] were present with highest levels in TCHZ. The SQDG(16:0_18:2) in TC4 (5.49 ± 0.27 μg/g) was lowest compared to TCHZ (13.36 ± 0.81 μg/g) and TC1 (11.21 ± 1.27 μg/g). PS(16:0_18:1) displayed the lowest levels in TCHZ, and the highest level in TC4. 2 PIs [PI(18:1_18:1) and PI(18:0_18:1)] were most abundant in TC1. 4 PEs [PE(20:1_18:2), PE(18:2_22:1), PE(18:3_18:1), and PE(18:2_22:1)] exhibited the highest levels in TC1, among which PE(20:1_18:2) (92.56 ± 0.77 μg/g) and PE(18:2_22:1) (10.94 ± 0.25 μg/g) were obviously higher than those in TCHZ and TC4. 2 PCs [PC(16:1_18:1), PC(20:1_18:2)] displayed higher content in TC1, and PC(16:0_16:0) exhibited higher level in TCHZ. PA(18:1_22:6) reached its peak content in TC1 (11.64 ± 1.65 μg/g), which was 2.05-fold and 2.77-fold higher than those in TC4 and TCHZ, respectively. 3 LPCs [LPC(18:1), LPC(18:2), LPC(18:0)] exhibited higher expression levels in TC4, which were significantly upregulated compared with TCHZ. The highest FFAs content was observed in TC4, with FFA(18:1), FFA(18:2), and FFA(20:1) being the primary differential FFAs, showing contents of 785.74 ± 45.83 μg/g, 65.29 ± 5.69 μg/g, and 14.48 ± 0.91 μg/g, respectively (Fig. 5B). FFA(18:1) and FFA(18:2) were evidently up-regulated in TC4, compared to TC1 and TCHZ.

Among TC1, TC4, and TCHZ samples, 3 common differential lipids were identified (Fig. 5C). Notably, PE(18:1_24:1) demonstrated the highest content in TC1, with progressively lower levels in TC4 and TCHZ (Fig. 5D), establishing it as a distinctive lipid marker among the 3 sample groups. To further elucidate the lipid metabolic differences among TC samples from different geographical regions, a comparative analysis was conducted by mapping the identified differential lipids to the KEGG database. Our results revealed significant associations between geographical origin and 5 key metabolic pathways, namely biosynthesis of unsaturated fatty acid, glycosylphosphatidylinositol (GPI)-anchor biosynthesis, glycerophospholipid metabolism, glycerolipid metabolism, and linoleic acid metabolism (Fig. 5E). Among these, glycerophospholipid metabolism exhibited the strongest correlation with geographical origin, followed by glycerolipid metabolism.

### 3.6 The differential lipids among TS, GN, and ZJ samples

**Fig. 6:**
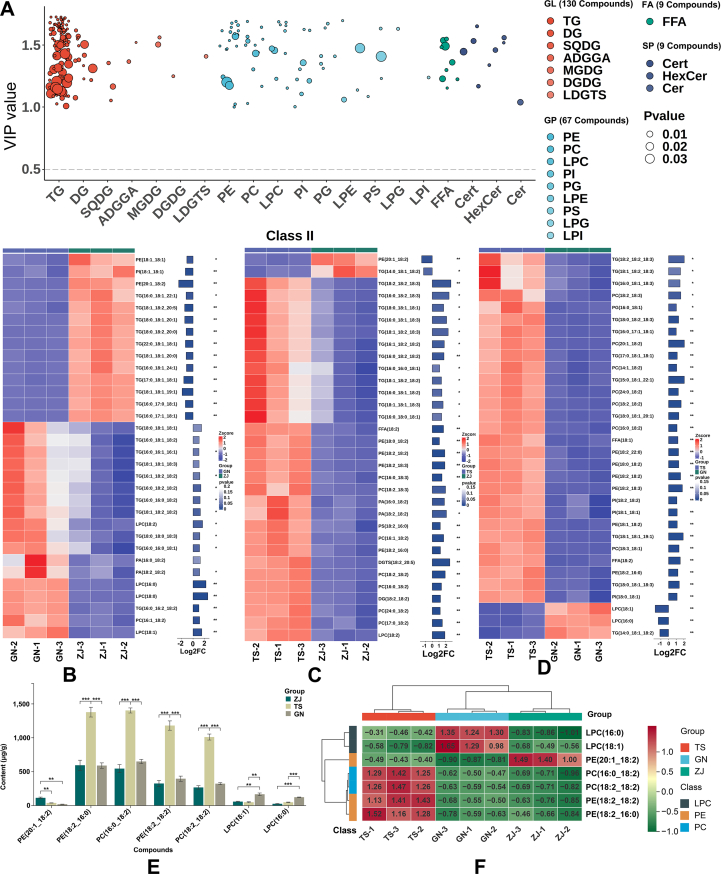
**A:** Bubble plot of the differential lipids among ZJ, GN, and TS. **B-D:** The combined heatmap of differential lipids in GN vs ZJ comparison, TS vs ZJ comparison, and TS vs GN comparison. **E:** Two sets of test histograms. **F:** The heatmap clustering of 7 differentially lipids.

Comparative lipidomic profiling of TS, GN, and ZJ samples revealed substantial variations in lipid content, with 215 differentially lipids identified across 4 major classes (**Table S11**). These comprised 9 SPs, 67 GPs, 130 GLs, and 9 FAs. Further subclass analysis displayed predominant representation by 106 TGs, 21 PEs, 15 DGs, 12 PCs, 10 LPCs, and 9 FFAs (Fig. 6A). Functional enrichment analysis indicated their primary involvement in glycerolipid metabolism and glycerophospholipid metabolism pathways.

In the GN_vs_ZJ comparison, 169 differentially lipids were detected (113 downregulated, 56 upregulated) (**Table S12**), among which 32 differentially lipids with higher levels were displayed in Fig. 6B. Notably, GN samples exhibited significantly elevated levels of 6 TGs compared to ZJ samples, including TG(16:0_18:1_18:2) (2.09-fold), TG(18:0_18:1_18:1) (2.80-fold), TG(18:1_18:2_18:2) (2.41-fold), TG(16:0_18:2_18:2) (2.56-fold), TG(18:1_18:1_18:3) (2.07-fold), TG(16:0_16:0_18:1) (2.19-fold). Conversely, ZJ samples exhibited distinct enrichment of TG(17:0_18:1_18:1), TG(18:0_18:2_20:0), and TG(18:1_18:1_20:0). Among GPs, the levels of 4 LPCs [LPC(18:1), LPC(16:0), LPC(18:2), and LPC(18:0)] were significantly higher than those in GN samples, whereas PE(18:1_18:1), PE(20:1_18:2), and PI(18:1_18:1) were significantly enriched in ZJ samples.

For the TS_vs_ZJ comparison, 190 differentially expressed lipids were identified (137 downregulated, 53 upregulated) (**Table S13**), with 32 elevated differential lipids illustrated in Fig. 6C. TS samples demonstrated significant enrichment of 5 TGs, comprising TG(16:0_18:1_18:2) (2.69-fold), TG(16:0_18:0_18:1) (2.64-fold), TG(18:0_18:1_18:1) (3.23-fold), TG(18:1_18:1_18:3) (3.04-fold), and TG(16:0_18:2_18:2) (3.35-fold). Additionally, PE(18:2_16:0), PE(18:2_18:2), PC(18:2_18:2), and PC(16:0_18:2) were markedly higher in TS samples, while PE(20:1_18:2) was obviously higher in ZJ samples.

In the TS_vs_GN comparison, 215 differentially expressed lipids were identified (187 downregulated, 28 upregulated) (**Table S14**), with 19 GPs, 11 GLs, and 2 FFAs showing higher abundance (Fig. 6D). Among GPs, 3 PEs [PE(18:2_16:0), PE(18:2_18:2), and PE(18:1_18:2)] displayed higher accumulation in TS samples, with 2.33-, 2.978-, and 2.20-fold increases compared to ZJ samples, respectively. Similarly, 2 PCs [PC(16:0_18:2), PC(18:2_18:2)] and 2 PIs [PI(18:1_18:1), PI(18:0_18:1)] were significantly elevated in TS samples. In contrast, 2 LPCs [LPC(18:1) and LPC(16:0)]were more abundant in GN samples. The 2 FFAs, FFA(18:1) and FFA(18:2), were notably higher in TS samples. Among GLs, 3 TGs [TG(18:0_18:2_18:3), TG(16:0_18:1_18:3), and TG(17:0_18:1_18:1)] were present with high levels in TS samples, while TG(14:0_18:1_18:2) were predominated in GN samples.

In summary, the lipidomic analysis revealed distinct profiles among the 3 samples, with significantly higher levels of LPC(18:1) and LPC(16:0) in GN samples compared to both ZJ and TS samples (Fig. 6E). Notably, TS samples exhibited the highest concentrations of PE(18:2_16:0), PC(16:0_18:2), PE(18:2_18:2), and PC(18:2_18:2). In contrast, ZJ samples showed a unique lipid signature, containing the highest abundance of PE(20:1_18:2) among all groups. The heatmap analysis further revealed that the 7 differentially lipids displayed statistically significant variations among the 3 samples, with clear clustering into 3 distinct groups, indicating their characteristic role as characteristic differential lipids (Fig. 6F).

### 3.7 Identification of lipid biomarkers across those 11 ROCs samples

**Fig. 7:**
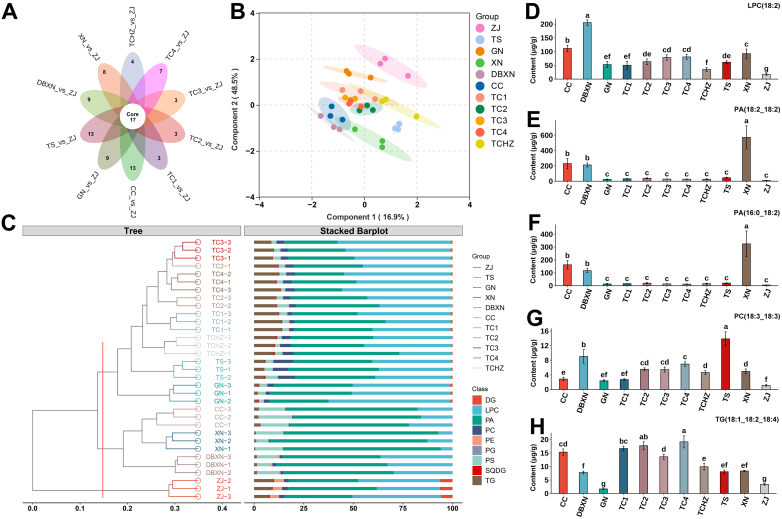
**A**: The Veen plot of 10 comparison. **B**: OPLS-DA score plot based on the 17 common differential lipids. **C**: Cluster bar plot. **D-H:** Comparison of the contents of LPC(18:2), PA(18:2_18:2), PA(16:0_18:2), PC(18:3_18:3), and TG(18:1_18:2_18:4) across of 11 ROCs, respectively.

Using ZJ samples as controls, 17 common differentially expressed lipids were consistently identified across all 10 comparative groups (Fig. 7A). To enhance separation between different ROCs groups and further explore differential lipids, supervised OPLS-DA analysis was performed. The OPLS-DA model demonstrated effective classification of 11 samples into three distinct clusters: Cluster I contained exclusively ZJ samples; Cluster II encompassed CC, XN, and DBXN groups; while Cluster III comprised TC lines along with TS and GN. Notably, Cluster III could be further stratified into TC lines, TS, and GN subgroups, indicating finer lipids metabolic differentiation (Fig. 7B). Cluster validation through bar plot analysis revealed clear tripartite separation at a 0.1 distance threshold (Fig. 7C), with hierarchical clustering patterns consistent with OPLS-DA results. This concordance confirmed the 17 differential lipids as robust biomarkers distinguishing the 11 ROCs.

Among the 17 common differential lipids, LPC(18:2), PA(18:2_18:2), PA(16:0_18:2), PC(18:3_18:3), and TG(18:1_18:2_18:4) exhibited higher contents compared to other differential substances. Multi-group comparison bar plots demonstrated that CC, XN, and DBXN exhibited the highest LPC(18:2) levels, with TX lines, TS, and GN displaying significantly elevated LPC(18:2) content relative to ZJ (Fig. 7D). Both PA(18:2_18:2) and PA(16:0_18:2) displayed obviously enrichment in CC, XN, and DBXN relative to other ROCs (Fig. 7E-F). PC(18:3_18:3) exhibited significantly greater content in TS compared to other groups (Fig. 7G), while TG(18:1_18:2_18:4) showed higher levels in the TC lines (Fig. 7H).

## 4 Discussion

The application of UPLC-MS/MS in lipidomics had significantly enhanced the coverage and sensitivity of lipid detection (Castañeda et al., 2024; Li et al., 2020). Lipidomics profiling utilizing UPLC-MS/MS have been successfully applied to characterize lipid compositions across multiple oil varieties, such as olive oil (Shen et al., 2013), flaxseed oil (Zhang et al., 2021), peanut oil (Zhang, Guo, et al., 2022), *Brassica napus* seeds (Woodfield et al., 2017) and hazelnut oil (Sun et al., 2022). The quantitative lipidomics approach had successfully investigated the lipid composition of 23*C. oleifera* lines in the southern Shaanxi and established a metabolic profile of lipid accumulation in *C. oleifera* from the Qinba Mountains area, thereby providing a solid data foundation for breeding distinctive *C. oleifera* varieties (Zheng et al., 2024). The present study provided a comprehensive comparison of the lipid profiles and nutritional quality of 11 ROCs, utilizing lipidomics and chemometrics approaches. 618 lipids were identified, categorized into 8 major classes, with TGs, PCs, PEs, PIs, DGs, and FFAs as the predominant lipid subclasses. Other subclasses, including PAs, PSs, MGs, LPCs, and LPEs, were also identified with significantly higher content.

This extensive lipid identification significantly enhanced the understanding of lipid composition in ROCs, establishing a foundation for better utilization of lipids. The chain length and degree of unsaturation of lipids influenced their structural and functional properties, thereby affecting digestive performance (Alves et al., 2022; Cui, Wang, et al., 2024). The results demonstrated that the carbon chain lengths of lipids in ROCs were concentrated within 32–58, classifying them as medium- and long-chain lipids, a characteristic closely associated with energy metabolism regulation, which might achieve weight management by promoting energy expenditure and reducing fat accumulation (Costa et al., 2024). Notably, TC lines, DBXN, and TS samples exhibited particularly higher proportions of medium- and long-chain lipids, suggesting their critical application values in developing low-calorie functional food formulations (Hopiavuori et al., 2019). Unsaturated lipids, essential human nutrients, played vital roles in blood lipid regulation, antithrombosis, and atherosclerosis prevention (Qin et al., 2024). ROCs demonstrated an 85.6 % unsaturation rate, with TCHZ, DBXN, TC1, and TC4 showing higher unsaturated lipid content, making them potentially effective in preventing coronary heart disease, cholesterol disorders, and diabetes (Yang et al., 2023). Importantly, significant heterogeneity in lipid composition existed among different samples, and this diversity directly impacted functional characteristics (Jiang et al., 2025; Koopman, 2018). DGs, through unique metabolic pathways, reduced body fat accumulation. The TC lines exhibited the highest DGs content (above 1820 μg/g), indicating potential applications in visceral fat reduction (Gao et al., 2022). GPs, crucial health-related compounds, were essential for maintaining basic physiological activities and enhancing immunity (Zhang et al., 2019). TC1, TC2, TC3, TS, and TCHZ samples contained higher GPs, with the content of 13,458.72 ± 782.98 μg/g, 12,782.11 ± 410.36 μg/g, 12,969.72 ± 446.11 μg/g, 13,481.92 ± 190.96 μg/g, and 12,301.15 ± 328.23 μg/g, respectively, demonstrating potential for promoting brain health, cardiovascular protection, and liver protection. Particularly noteworthy were CC and TC4 samples, whose higher FFA(18:1) content played a pivotal role in cardiovascular disease prevention, neurodevelopment regulation, and maintaining inflammatory response balance (Teres et al., 2008). These findings collectively established an integrative framework that elucidated both the diversity of lipid components and their associated biological functions in ROCs.

Previous investigations have predominantly emphasized the identification of trace lipid components in *C. oleifera*, including FFAs and FFA(18:1) (Peng et al., 2024; Wei et al., 2022). However, comprehensive studies employing large-scale absolute quantitative profiling to elucidate lipid composition variations across diverse ROCs remained notably scarce. In this study, chemometrics analysis uncovered substantial heterogeneity in lipid composition and content across 11 ROCs, with 11 distinct samples divided into 3 categories. The inter-sample variability in lipid compositional content observed in this study displayed substantial amplitude, indicative of pronounced genetic diversity. These findings could potentially stem from geographical disparities, cultivar-specific characteristics, and analogous influential determinants. The category I (Wuling Mountain regions: CC, XN, DBXN) demonstrated 202 significantly differentiated lipids, predominantly enriched in GLs, GPs, and FFAs. Notably, CC exhibited markedly elevated contents of FFA(18:1) and FFA(18:2) compared to XN and DNXN, yet showed lower PE(20:1_18:2) content. DBXN displayed the highest content of TGs, including TG(16:0_18:1_18:2), TG(18:0_18:1_18:1), TG(16:0_18:2_18:2), TG(18:1_18:2_18:2), TG(18:1_18:1_18:3), and TG(16:0_18:0_18:1), whereas XN exhibited highest PA(18:2_18:2) content. PE(20:1_18:2), FFA(20:1), and FFA(22:1) were identified as distinctive biomarkers differentiating the 3 ROCs. The category II was composed of TC series, among which there were significant differences between TC1, TC4 and TCHZ. The differential lipids among TC1, TC4 and TCHZ were mainly TGs, PIs, FFAs, PEs and PCs. TC1 exhibited the highest PE(20:1_18:2), while TC4 had elevated FFA(18:1) and FFA(18:2) compared to TC1 and TCHZ. Conversely, TCHZ exhibited pronounced increases in TG(14:0_18:1_18:2), TG(16:0_16:2_18:2), and TG(16:0_16:1_16:1). The category III (ZJ, TS, GN) exhibited differential lipid profiles primarily involving TGs, PEs, DGs, PCs, LPCs, and FFAs. TS displayed higher contents of PE(18:2_16:0), PC(16:0_18:2), PE(18:2_18:2), and PC(18:2_18:2) compared to ZJ and GN. ZJ exhibited maximal PE(20:1_18:2) levels, whereas GN showed elevated LPC(18:1) and LPC(16:0). Using ZJ sample as a control, 17 common differential lipids were identified across 10 comparison groups. Among them, 5 characteristic differential lipids, LPC(18:2), PA(18:2_18:2), PA(16:0_18:2), PC(18:3_18:3), and TG(18:1_18:2_18:4), were ultimately selected as biomarkers with higher content for the 11 ROCs, which were enriched in glycerolipid metabolism and glycerophospholipid metabolism pathways. Our investigation enhanced the understanding of ROCs lipid profiles, provided a foundation for constructing potential biomarker sets, evaluating ROC quality, and developing functional food applications.

**Fig. 8:**
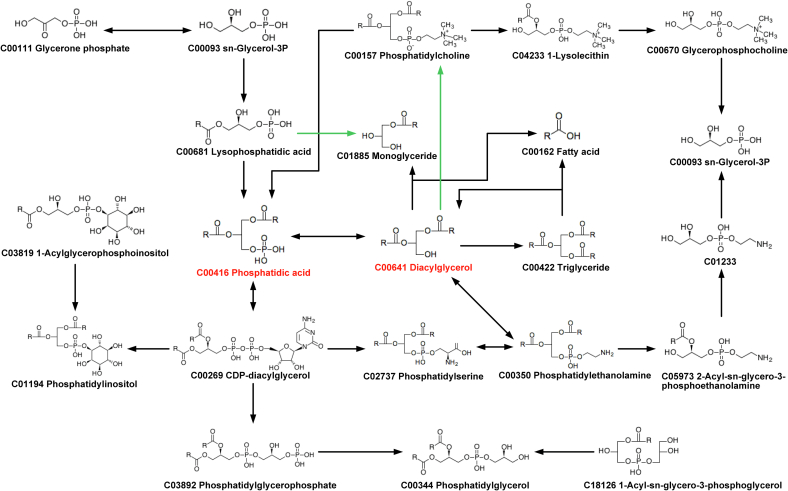
Overview of key lipid metabolic pathways (glycerophospholipid metabolism and glycerolipid metabolism) in 11 ROCs.

During the ripening progression of ROCs, the lipid metabolic network demonstrated dynamic regulatory features characterized by interconnected metabolic pathways. Based on the differential lipids across 11 ROCs and the KEGG pathway, the core regulatory nodes of 11 ROCs comprised DGs, TGs, FFAs, PAs, PCs, PEs, and PIs (Fig. 8). PAs emerged as the central metabolic precursor, undergoing dephosphorylation to generate PCs, PEs, and PIs species, thereby establishing the structural framework for GPs biosynthesis (Haslam et al., 2016). Simultaneously, these phospholipid species facilitated dynamic membrane remodeling while performing multiple biological roles including membrane integrity maintenance, signal transduction modulation, cellular physiology coordination, and energy metabolism homeostasis (Liu et al., 2023).Functioning as direct biosynthetic precursors for both TGs and FFAs, DGs served dual roles as structural units for GLs assembly and critical regulators of membrane organization, energy storage dynamics, signaling cascades, and metabolic equilibrium (Cao et al., 2023; Lu et al., 2009). Notably, PAs and DGs were the core differential lipids, and metabolic interconversion between DGs and PAs was observed under specific conditions, creating a biochemical bridge between glycerophospholipid and glycerolipid metabolic pathways. This cross-pathway regulation originated from a shared enzymatic initiation point: glycerol-3-phosphate acyltransferase catalyzed the primary conversion of glycerol-3-phosphate to LPAs (Huang et al., 2022). This bidirectional metabolic regulatory mechanism persisted throughout the entire fruit ripening process, constituting the core regulatory characteristic of the lipid metabolic network in ROCs.

Our findings revealed significant variations in lipid composition and nutritional attributes, and offered valuable insights for optimizing its production and application in the food and health industries. However, the mechanistic links between these lipid species and specific health outcomes required systematic investigation. Targeted studies integrating multi-omics approaches (lipidomics, metabolomics, proteomics) with isotope tracing studies are warranted to elucidate the complete metabolic circuits and assess their therapeutic potential or dietary implications.

## 5 Conclusion

In this study, comprehensive lipid compositions and contents variations across 11 ROCs were determined using lipidomics based on UPLC-MS/MS. A total of 618 lipids were identified, with TGs, PCs, PEs, PIs, DGs, and FFAs as the predominant lipid subclasses. The highest contents of total lipids and GLs were observed in TC1, followed by TC4 and DBXN. GPs were significantly elevated in TC1, TC2, TC3, and TS, while CC samples displayed the highest FFAs content. The nutritional quality assessment revealed that CC, TS, DBXN, and TC lines exhibited a unique lipid profile, making them promising candidates for development into novel functional food products with enhanced nutritional value. Chemometric analysis of 11 ROCs revealed distinct clustering into 3 categories: CC-DBXN-XN, TC lines, and ZJ-GN-TS. Using ZJ as a control, LPC(18:2), PA(18:2_18:2), PA(16:0_18:2), PC(18:3_18:3), and TG(18:1_18:2_18:4) were recognized as biomarkers for distinguishing the 11 ROCs. The KEGG pathway analysis of these differentially accumulated lipids revealed glycerophospholipid and glycerolipid metabolism as the predominant regulatory pathways. Importantly, subsequent bioinformatics annotation further identified PAs, PCs, PEs, TGs, DGs, and FFAs as critical lipids metabolites involved in those 2 pathways. This study conducted a comprehensive characterization of the lipid compositions and functions in 11 ROCs, revealing distinct lipid profile variations, elucidating critical regulatory nodes in lipid biosynthesis pathways, which laid a solid foundation for the precision targeted breeding and developing functional food products of ROCs.

## CRediT authorship contribution statement

**Zhuang Deng:** Methodology, Data curation. **Jianmei Yang:** Validation, Resources, Investigation. **Xinye Zhang:** Resources, Investigation. **Chunyao Dun:** Resources, Investigation. **Zhubing Hu:** Software, Methodology. **Guodong Wang:** Supervision, Methodology. **Qi Tang:** Investigation, Data curation. **Tao Zheng:** Writing – review & editing, Investigation, Funding acquisition. **Haitao Zeng:** Project administration, Investigation, Funding acquisition, Conceptualization.

## Declaration of competing interest

All authors declared that they had no known competitive financial interests or personal relationships that could have appeared to influence the study reported in this paper.

## Data Availability

All the lipids data of 11 red-flowered oil-tea camellia demonstrated in this study were availably obtained on request.

## Appendix A Supplementary data

Supplementary material 1 Fig.S1. Mass spectrometry analysis of MRM detection of multimodal map. Fig.S2. The total ion current curves. Supplementary material 1

Supplementary material 2 The lipids internal standard. Supplementary material 2

Supplementary material 3 618 lpids identified across 11 ROCs. Supplementary material 3

Supplementary material 4 Differential lipids among XN, DBXN, and CC samples. Supplementary material 4

Supplementary material 5 The differential lipids in the CC_vs_XN comparison. Supplementary material 5

Supplementary material 6 The differential lipids in the CC_vs_DBXN comparison. Supplementary material 6

Supplementary material 7 The differential lipids in the DBXN_vs_XN comparison. Supplementary material 7

Supplementary material 8 191 differential lipids across TC lines. Supplementary material 8

Supplementary material 9 The differential lipids in the TC4_vs_TC1 comparison. Supplementary material 9

Supplementary material 10 The differential lipids in the TCHZ_vs_TC1 comparison. Supplementary material 10

Supplementary material 11 The differential lipids in the TCHZ_vs_TC4 comparison. Supplementary material 11

Supplementary material 12 The differential lipids among TS, GN and ZJ samples. Supplementary material 12

Supplementary material 13 The differential lipids in the GN_vs_ZJ comparison. Supplementary material 13

Supplementary material 14 The differential lipids in the TS_vs_ZJ comparison. Supplementary material 14

Supplementary material 15 The differential lipids in the TS_vs_GN comparison. Supplementary material 15
