# Supplementary material for: Comparison of lipid profiles and nutritional quality of 11 red-flowered oil-tea camellia through quantitative lipidomics and chemometrics
Source: Food Chem X. 2025 Jul 4;29:102738. doi: 10.1016/j.fochx.2025.102738 (PMC12275141; doi:10.1016/j.fochx.2025.102738)
Supplement: Supplementary material 1 — Fig.S1. Mass spectrometry analysis of MRM detection of multimodal map. Fig.S2. The total ion current curves. [file mmc1.docx]

**Fig.S1**. **A**: Mass spectrometry analysis of MRM detection of multimodal map-P. **B**: Mass spectrometry analysis of MRM detection of multimodal map-P.





**Fig.S2**. **A**: The total ion current curves of the positive ion mode (P+). **B**: The total ion current diagram of the negative ion mode (N-).
